# Integrative analysis of transcriptomes highlights potential functions of transfer-RNA-derived small RNAs in experimental intracerebral hemorrhage

**DOI:** 10.18632/aging.103938

**Published:** 2020-11-16

**Authors:** Peng-Fei Li, Shi-Chao Guo, Tao Liu, Hanjin Cui, Dandan Feng, Ali Yang, Zhe Cheng, Jiekun Luo, Tao Tang, Yang Wang

**Affiliations:** 1 Institute of Integrative Medicine, Department of Integrated Traditional Chinese and Western Medicine, Xiangya Hospital, Central South University, Changsha 410008, China; 2 Department of Respiratory and Critical Care Medicine, The First Affiliated Hospital of Zhengzhou University, Zhengzhou 450052, China; 3 Henan Key Laboratory for Pharmacology of Liver Diseases, Institute of Medical and Pharmaceutical Sciences, Zhengzhou University, Zhengzhou 450052, China; 4 Department of Neurosurgery, The First Affiliated Hospital of Zhengzhou University, Zhengzhou 450052, China; 5 Department of Gerontology, Traditional Chinese Medicine Hospital Affiliated to Xinjiang Medical University, Urumqi 830011, China; 6 Department of Neurology, Henan Province People’s Hospital, Zhengzhou 450003, China

**Keywords:** intracerebral hemorrhage, rats, transcriptome, non-coding RNA, transfer-RNA-derived small RNA

## Abstract

Transfer-RNA-derived small RNAs (tsRNAs) are a novel class of short non-coding RNAs, that possess regulatory functions. However, their biological roles in hemorrhagic stroke are not understood. In this study, by RNA sequencing, we investigated the tsRNA expression profiles of intracerebral hemorrhagic rat brains in the chronic phase. A total of 331 tsRNAs were identified (308 in sham and 309 in intracerebral hemorrhage). Among them, the validation revealed that 7 tsRNAs (1 up-regulated and 6 down-regulated) were significantly changed. Subsequently, we predicted the target mRNAs of the 7 tsRNAs. Through integrative analysis, the predicted targets were validated by mRNA microarray data. Moreover, we confirmed the functions of tsRNAs targeting mRNAs *in vitro*. Furthermore, using bioinformatics tools and databases, we developed a tsRNA-mRNA-pathway interaction network to visualize their potential functions. Bioinformatics analyses and confirmatory experiments indicated that the altered genes were mainly enriched in several signaling pathways. These pathways were interrelated with intracerebral hemorrhage, such as response to oxidative stress, endocytosis, and regulation of G protein-coupled receptor signaling pathway. In summary, this study systematically revealed the profiles of tsRNAs after an experimental intracerebral hemorrhage. These results may provide novel therapeutic targets following a hemorrhagic stroke in the chronic phase.

## INTRODUCTION

Intracerebral hemorrhage (ICH) is a particularly lethal form of stroke, with an estimated mortality rate of approximately 40% within 1 month [1, 2]. Furthermore, 80% of survivors cannot perform activities of daily living during the first 6 months, remaining significantly disabled in the long term [1, 3]. With the advancement of early diagnoses and decompressive craniectomy, mortality rates of ICH in the acute phase have decreased significantly [4, 5]. Afterward, the problem of functional recovery in the chronic phase becomes dominant in the treatment of ICH [6]. However, neither the disability rate nor the long-term life quality of survivors has improved markedly [6]. Consequently, a deeper understanding of the pathophysiology after ICH is of pivotal importance for developing new therapeutic approaches that can promote functional recovery.

Previous studies have demonstrated that non-coding RNAs (ncRNAs), accounting for 98% of total RNAs, are the important regulators of various molecular processes [7, 8]. For instance, microRNAs (miRNAs) and long ncRNAs that control the post-transcription of protein-coding gene expression, play fundamental roles in many pathological mechanisms of hemorrhagic stroke [9–12]. Due to the complications of ICH, there is an urgent need to identify more functional ncRNAs involved in the pathological process. With the recent advances in small RNA sequencing, a novel class of short ncRNA has been discovered, known as transfer-RNA-derived small RNA (tsRNA, less than 50 nucleotides, nt) [13–15]. There are at least six types of tsRNA that differ in the cleavage position of the precursor or mature tRNA transcript (Supplementary Figure 1A, 1B) [16]. The tRNA-derived fragment 1 (tRF-1) is generated from the uracil rich trailer sequence upstream of the precursor tRNA [17]. As a consequence of cleavages in different loops of the mature tRNAs, tRFs are classified into tRF-5 (5’ to D-loop), tRF-3 (T-loop to 3’), and internal tRF (i-tRF) [17]. Similarly, the cleave of anticodon-loops of mature tRNAs cleaves tRNA into two subtypes of stress-induced tRNA fragments (tiRNA): tiRNA-5 (5’ to anticodon-loop) and tiRNA-3 (anticodon-loop to 3’) [18].

tsRNAs are extremely widespread in most organisms and have been implicated in stress responses, cancer, viral infection responses, and neurological disorders, Nevertheless, their biological roles are still not well understood [16, 17, 19–21]. Ongoing studies have suggested that tsRNAs are similar to miRNAs in length and structure, suggesting a miRNA-like function of tsRNAs [17, 18]. The tsRNAs contain some seed sequences that might match the seed regions of mRNA by antisense pairing, regulating the expression level of target mRNAs [22–24]. Thus, tsRNAs might function as important regulatory molecules in the pathogenesis of diseases. Moreover, recent studies have confirmed that tsRNAs play a pivotal role in aging and cerebral ischemia [20, 25]. In our previous study, we found that tsRNAs might be potential therapeutic targets for ICH treatment [26]. Unfortunately, the systemic functions of tsRNAs in hemorrhagic stroke are still unclear.

**Figure 1:**
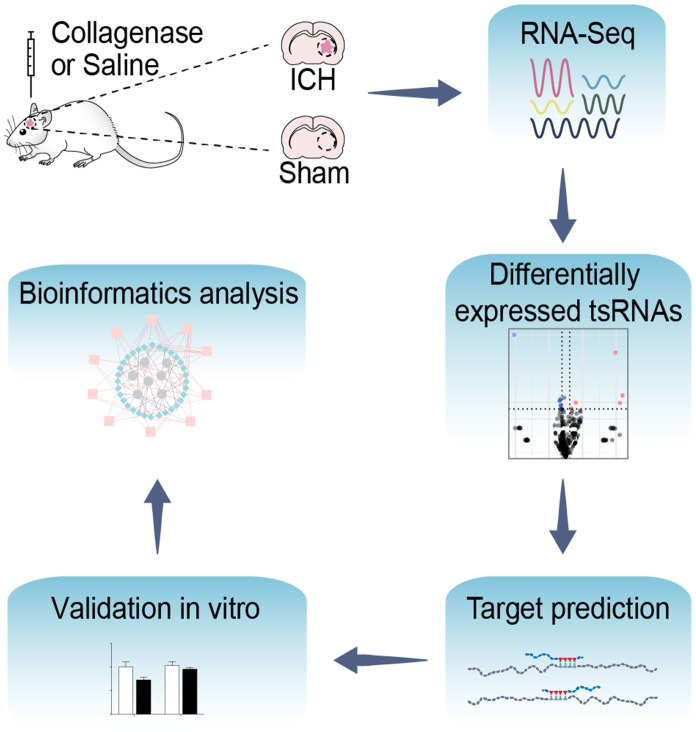
**Flow chart of the present study.** This study was designed as follows: rat brain tissues of ICH and sham were collected and then altered expressed tsRNAs with altered expression were obtained by RNA-Sequencing. Subsequently, we predicted and validated the mRNA targets of ICH-responsive tsRNAs. Furthermore, we evaluated the potential tsRNA functions post-ICH by bioinformatics tools. Finally, the bioinformatics results were validated *in vitro*.

Thus, to access the roles of the novel ncRNA post-ICH in the chronic phase, we explored the tsRNA expression profile in the rat brain tissue surrounding the hemorrhagic region. Using bioinformatics, we further identified potential mRNA targets and evaluated the putative biological functions of the ICH-responsive tsRNAs to reveal their potential functions in the pathophysiologic processes of ICH (the study design is shown in Figure 1).

## RESULTS

### tsRNA expression profile in ICH rats

We used RNA sequencing (RNA-Seq) to assess the tsRNA profiles in ICH rats relative to those in sham. The original data were submitted to Gene Expression Omnibus (GEO accession: GSE109697). Because there are no databases of rat tsRNAs yet, we used a uniform system for naming the rat tsRNAs to assigned the name of each tsRNA (Supplementary Figure 1C). This naming system is similar to that used for miRNA [27], and each tsRNA has a unique name that exhibits its basic characteristics such as the type, length, and organism.

**Figure 2:**
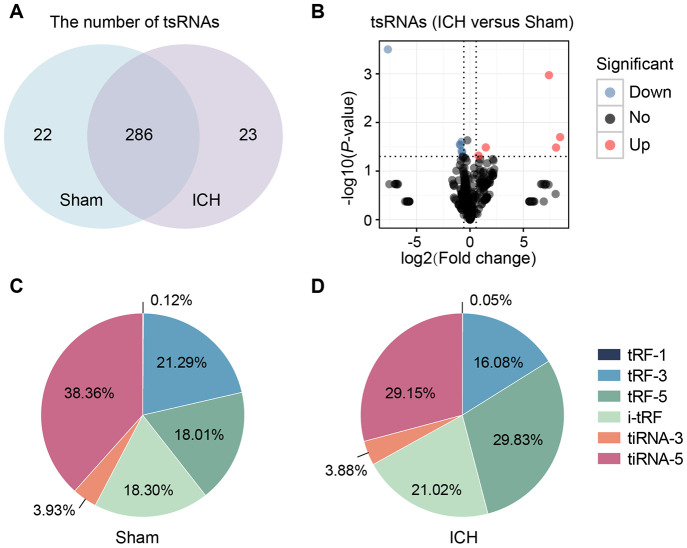
**tsRNAs were highly enriched in the ICH rat brain.** (**A**) Venn plot to show the identified tsRNA numbers in the brain tissues of sham and ICH. We totally identified 331 tsRNAs (308 in the sham group and 309 in the ICH). (**B**) Volcano plot of tsRNA comparison between the ICH and sham. The red points denoted five up-regulated tsRNAs and the blue ones denoted seven down-regulated ones (with the standard of fold change >1.5 and P<0.05). Percentage of each tsRNA type in total tsRNAs in the sham (**C**) and ICH (**D**) groups. TPM indicates the tag counts per million of total aligned tRNA reads, representing the tsRNA expression levels.

In this study, we identified a total of 331 tsRNAs (308 in the sham group and 309 in the ICH) from the right globus pallidus region of rat brains (Figure 2A, 2B and Supplementary Table 1). The content of the tsRNA was widely different among each type. The tsRNAs (tiRNA-5, tiRNA-3, tRF-5, tRF-3, and i-tRF), that were derived from mature tRNAs, were the most abundant (more than 99%) (Figure 2C, 2D). In sham brain tissue, 38.36% and 21.29% of the tsRNAs were tiRNA-5 and tRF-3. Respectively, tRF-1 was minimal (found in only 0.12%) (Figure 2C). At 21 days after ICH, tiRNA-5 and tRF-3 decreased by 9.21% and 5.21%, respectively, while tRF-5 increased by 11.82% (Figure 2D). Additionally, the tsRNAs were mainly generated from tRNAs transferring Arginine (Arg), Glutamine (Gln), Glutamic acid (Glu), Glycine (Gly) and Serine (Ser). The tsRNAs derived from tRNAs transferring Methionine (Met) increased markedly (Supplementary Figure 2A, 2B). Moreover, the lengths of the tsRNAs were concentrated in the ranges of 16-23, 29-36, and 49 nt (Supplementary Figure 2C).

### Confirmation of ICH-responsive tsRNAs

**Figure 3:**
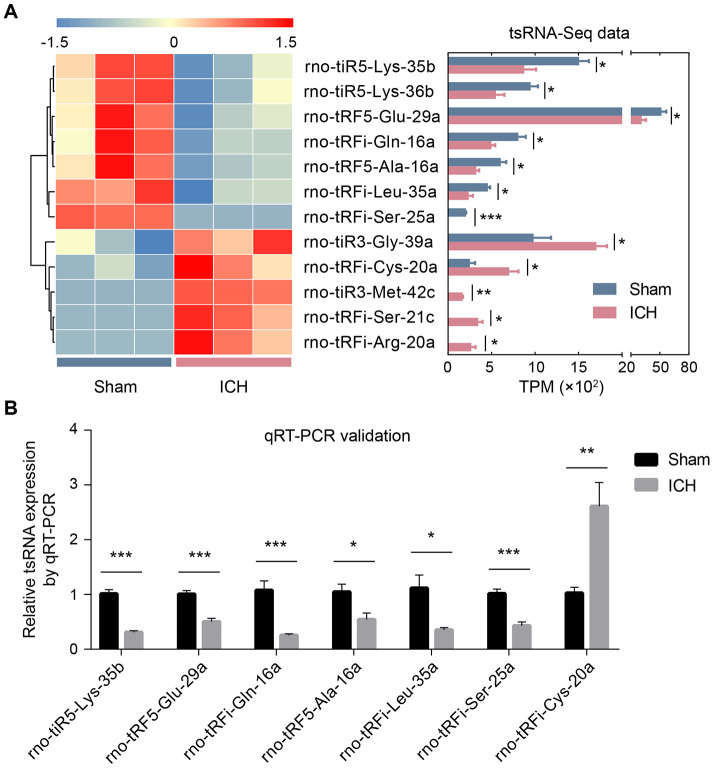
**The validation of ICH-responsive tsRNAs.** (**A**) The tsRNA-Seq data revealed 12 significantly changed tsRNAs. The 12 significantly changed tsRNAs are shown in the heat map (the left part of **A**). The expression levels of the 12 tsRNAs by RNA-sequencing data are exhibited in the bar chart (the right part of **A**). (**B**) The qRT-PCR results showed that among the 12 tsRNAs, only 7 tsRNAs were significantly changed. The 7 tsRNAs were confirmed as the actual ICH-responsive tsRNAs. Data are presented as the mean ± SEM, (n=3 each group in **A**, and n=6 each group in **B**), *P<0.05, ** *P*<0.01, *** *P*<0.001. TPM indicates tag counts per million of total aligned tRNA reads, representing the tsRNA expression levels.

With the standard of fold change >1.5 and *P* <0.05, we found 12 tsRNAs with significantly different expression levels in ICH rats compared with the sham (5 up-regulated and 7 down-regulated) (Figure 2B and Figure 3A). rno-tiR3-Gly-39a, rno-tRFi-Cys-20a, rno-tiR3-Met-42c, rno-tRFi-Ser-21c, and rno-tRFi-Arg-20a were significantly up-regulated, while rno-tiR5-Lys-35b, rno-tiR5-Lys-36b, rno-tRF5-Glu-29a, rno-tRFi-Gln-16a, rno-tRF5-Ala-16a, rno-tRFi-Leu-35a, and rno-tRFi-Ser-25a were significantly down-regulated.

The tsRNA-Seq data showed 12 significantly changed tsRNAs in the ICH rats. To validate these tsRNA-Seq results, we used qRT-PCR to confirm the expression changes of the tsRNAs in the rat brain tissues. The qRT-PCR results showed that among the 12 tsRNAs, there were 7 significantly changed tsRNAs (rno-tiR5-Lys-35b, rno-tRF5-Glu-29a, rno-tRFi-Gln-16a, rno-tRF5-Ala-16a, rno-tRFi-Leu-35a, rno-tRFi-Ser-25a. and rno-tRFi-Cys-20a) (Figure 3B). Moreover, the expression tendencies of the 7 tsRNAs between the qRT-PCR and tsRNA-Seq results were similar. Therefore, the 7 tsRNAs were confirmed as ICH-responsive tsRNAs and were subjected to further analysis.

### Integrative analysis of tsRNA and mRNA transcriptomes to validate the predicted targets of the ICH-responsive tsRNA*s*

**Figure 4:**
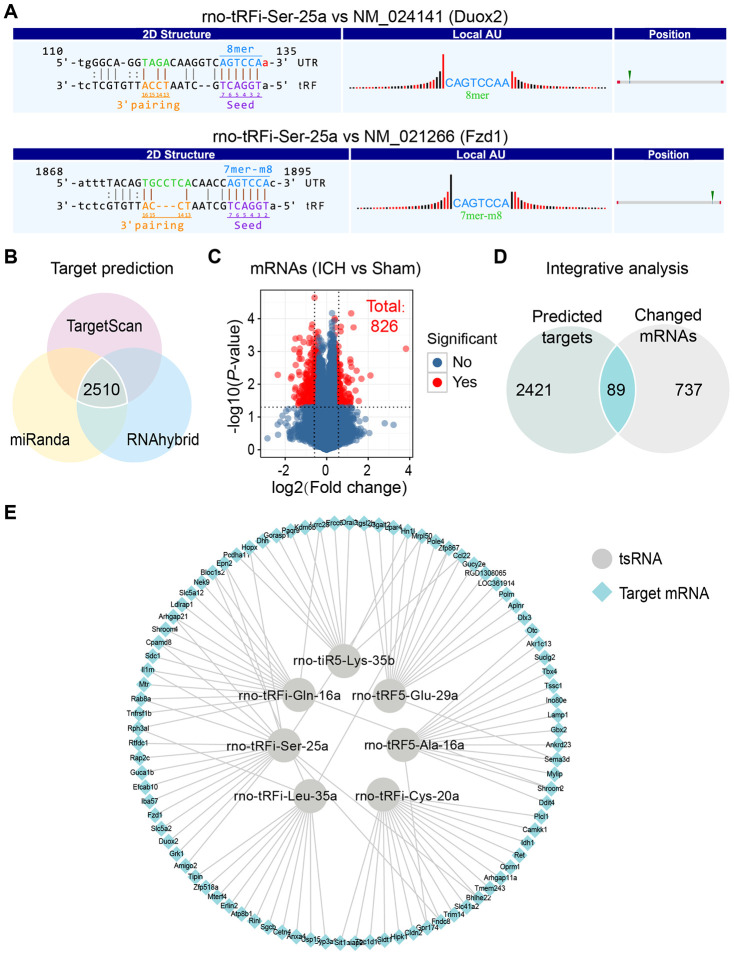
**Integrative analysis to confirm the targets of 7 ICH-responsive tsRNAs.** (**A**) The nucleotides of tsRNAs in the 5’ end could pair with the nucleotides of mRNAs in the 3’ end, which allow tsRNAs to target the mRNAs. The rno-tRFi-Ser-25a and its target mRNAs were selected to show the pairing method using TargetScan. (**B**) Venn plot indicating that 2510 target mRNAs of 7 ICH-responsive tsRNAs were predicted by 3 prediction algorithms simultaneously. (**C**) Volcano plot showing the mRNA profiles of the sham and ICH groups, among which 826 mRNAs were significantly changed (with the standard of fold change >1.5 and *P*<0.05). (**D**) The 2510 target mRNAs and 826 significantly changed mRNAs were integrated for integrative analysis, showing that 89 targets were significantly changed after ICH and might exert potential biological functions in ICH. (**E**) The 89 target mRNAs of 7 ICH-responsive tsRNAs are illustrated by Cytoscape software.

Increasing amounts of evidence has revealed that tsRNAs contain some seed sequences that might match the seed regions of mRNA by antisense pairing, regulating the expression level of the target mRNA [22–24] (Figure 4A). Although different algorithms can be used to get possible seed sequences and targets for tsRNAs such as TargetScan, RNAhybrid, RNA22 and so on [20, 28, 29], each methodology for tsRNA target prediction is referenced to miRNA target predictors. The nucleotides of tsRNAs in the 5’ end could pair with the nucleotides of mRNAs in the 3’ end, which allows tsRNAs to target the mRNAs. Here, we used 3 algorithms named RNAhybrid, TargetScan and miRanda to predict the mRNA targets of the 7 ICH-responsive tsRNAs. By applying the 3 algorithms together, we obtained a total of 2510 mRNA targets of the 7 tsRNAs (Figure 4B).

Considering that the target prediction results were acquired solely through bioinformatics methods, we needed to validate whether these mRNA targets existed in the brain tissues and how the expression levels of these targets were affected by tsRNAs. Hence, we used mRNA microarray for validation. In our previous study, we explored the mRNA expression levels in rat brain tissues at 21 days and identified 826 differentially expressed mRNAs (|log_2_ FC|>|log_2_ 1.5|, *P* <0.05) (Figure 4C and Supplementary Table 2) [30]. Then we integrated the 2510 mRNA targets and 826 differentially expressed mRNAs for integrative analysis to validate the predicted targets of the ICH-responsive tsRNAs. We found that 89 mRNA targets were contained in the 826 significantly altered mRNAs (Figure 4D and Supplementary Table 3). This result suggested that the 89 targets were significantly changed after ICH and might exert potential biological functions in ICH. Therefore, we used the 89 target mRNAs for subsequent bioinformatics analysis to reveal their functions. Each tsRNA and its target mRNAs were shown graphically (Figure 4E). Particularly, we found that one target mRNA could be bound by 2 tsRNAs. For instance, the mRNA Epn2 was targeted by rno-tRFi-Ser-25a and rno-tRFi-Gln-16a.

### Validating the functions of tsRNAs acting in a miRNA-like manner *in vitro*

The tsRNAs functioned in a miRNA-like manner, regulating the expression levels of mRNAs. Through the bioinformatics and microarray methods, we identified that the 7 ICH-responsive tsRNAs might target 89 mRNAs. To confirm the functions of these tsRNAs acting in a miRNA-like manner, we performed the cell transfection experiment and luciferase reporter assay in PC12 cells.

**Figure 5:**
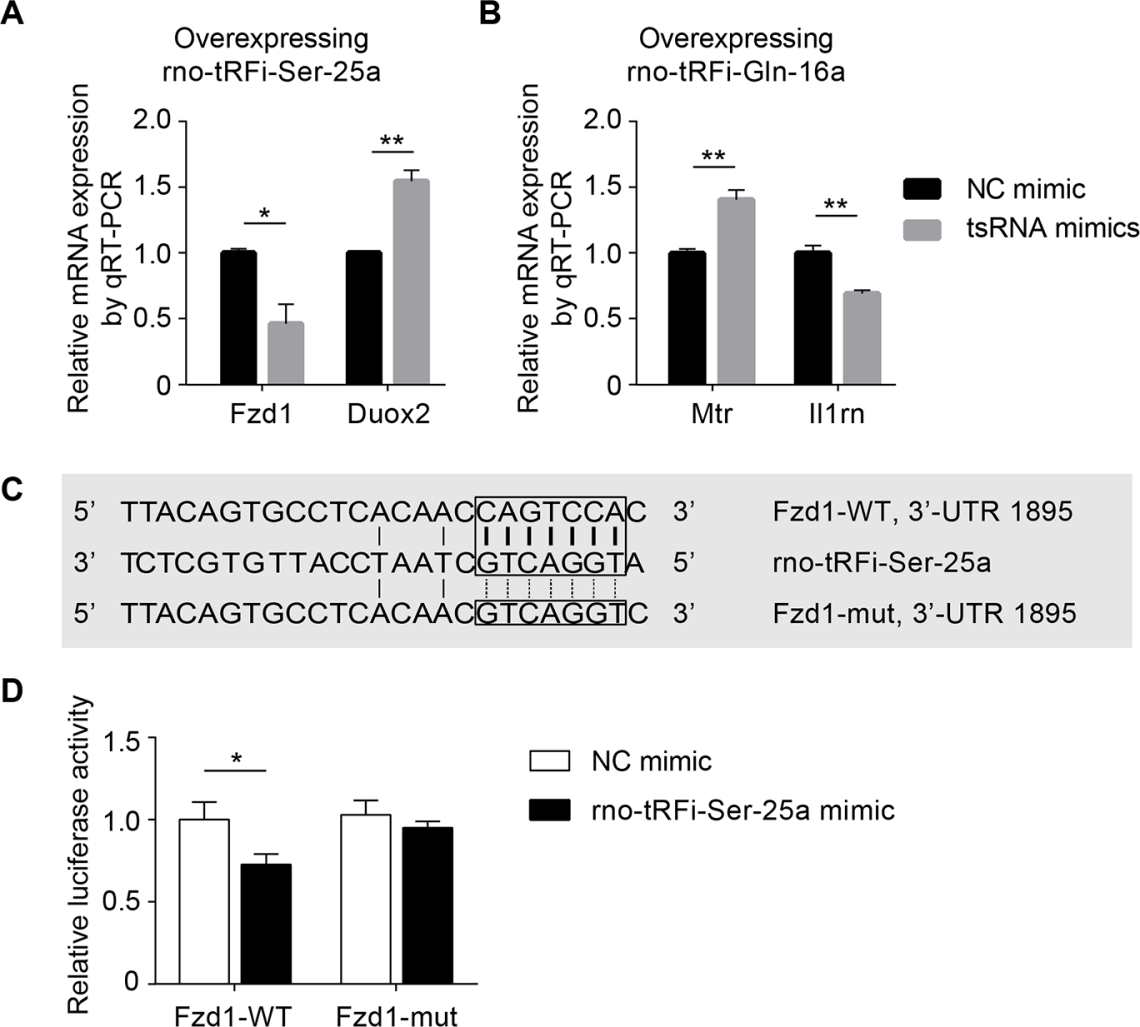
**Experiments *in vitro* to validate tsRNAs targeting mRNAs by complementarily pairing of the nucleotides.** (**A**) The relative Fzd1 and Duox2 levels were significantly changed after transfecting rno-tRFi-Ser-25a mimics in PC12 cells compared to the control (all *P* <0.05). (**B**) The relative expression levels of Mtr and Il1rn were significantly changed after transfecting rno-tRFi-Gln-16a mimics likewise. (**C**) Schematic representation of the potential binding sites for rno-tRFi-Ser-25a in the Fzd1 3’UTR. Seed sequences of the wild type (Fzd1-WT 3’UTR) and mutant type (Fzd1-mut 3’UTR) luciferase reporter showing the binding site. (**D**) The relative luciferase activity of the WT and mut reporter constructs, which were cotransfected with either the rno-tRFi-Ser-25a or negative control mimics. Data are presented as the ratio of luciferase activity from the negative control versus the rno-tRFi-Ser-25a mimic-transfected neurons. rno-tRFi-Ser-25a inhibited the luciferase activity of the WT, but not the mut reporter construct. rno-tRFi-Ser-25a directly targeted Fzd1 by binding to the 3’UTR sites. Data are presented as the mean ± SEM and *P<0.05, **P<0.01.

First, to validate that the tsRNAs could decrease the expression levels of the target mRNAs, we transfected tsRNA mimics into PC12 cells and detected the target mRNA levels by qRT-PCR. We randomly selected 2 tsRNAs and 2 target mRNAs (rno-tRFi-Ser-25a targeting Fzd1 and Duox2; rno-tRFi-Gln-16a targeting Mtr and Il1rn, respectively). After transfecting the rno-tRFi-Ser-25a, rno-tRFi-Gln-16a, and negative control mimics into PC12 cells, we detected the relative levels of Fzd1, Duox2, Mtr and Il1rn. The transfection experiment showed that compared to the control, the relative levels of the mRNA targets were significantly changed after transfecting rno-tRFi-Ser-25a or rno-tRFi-Gln-16a mimics (all *P* <0.05) (Figure 5A, 5B).

Subsequently, we searched for the regulatory functions of tsRNAs targeting mRNAs using luciferase reporter assays. rno-tRFi-Ser-25a and its target Fzd1 were randomly selected for verification. Then, we searched for the potential binding site in rno-tRFi-Ser-25a to the Fzd1 3’UTR using TargetScan (Figure 4A), and performed a luciferase reporter assay after cotransfecting PC12 cells with Fzd1 3’UTR constructs, containing the putative rno-tRFi-Ser-25a binding site with either the wild type (Fzd1 WT 3’UTR) or mutant type (Fzd1 Mut 3’UTR) (Figure 5C). We found that rno-tRFi-Ser-25a inhibited the luciferase activity of the WT, but not the Mut 3’UTR (Figure 5D). These results indicated that rno-tRFi-Ser-25a directly targeted Fzd1 and inhibited its expression by binding to the 3’UTR site.

### Bioinformatics analysis revealing potential biological functions of the tsRNAs

**Figure 6:**
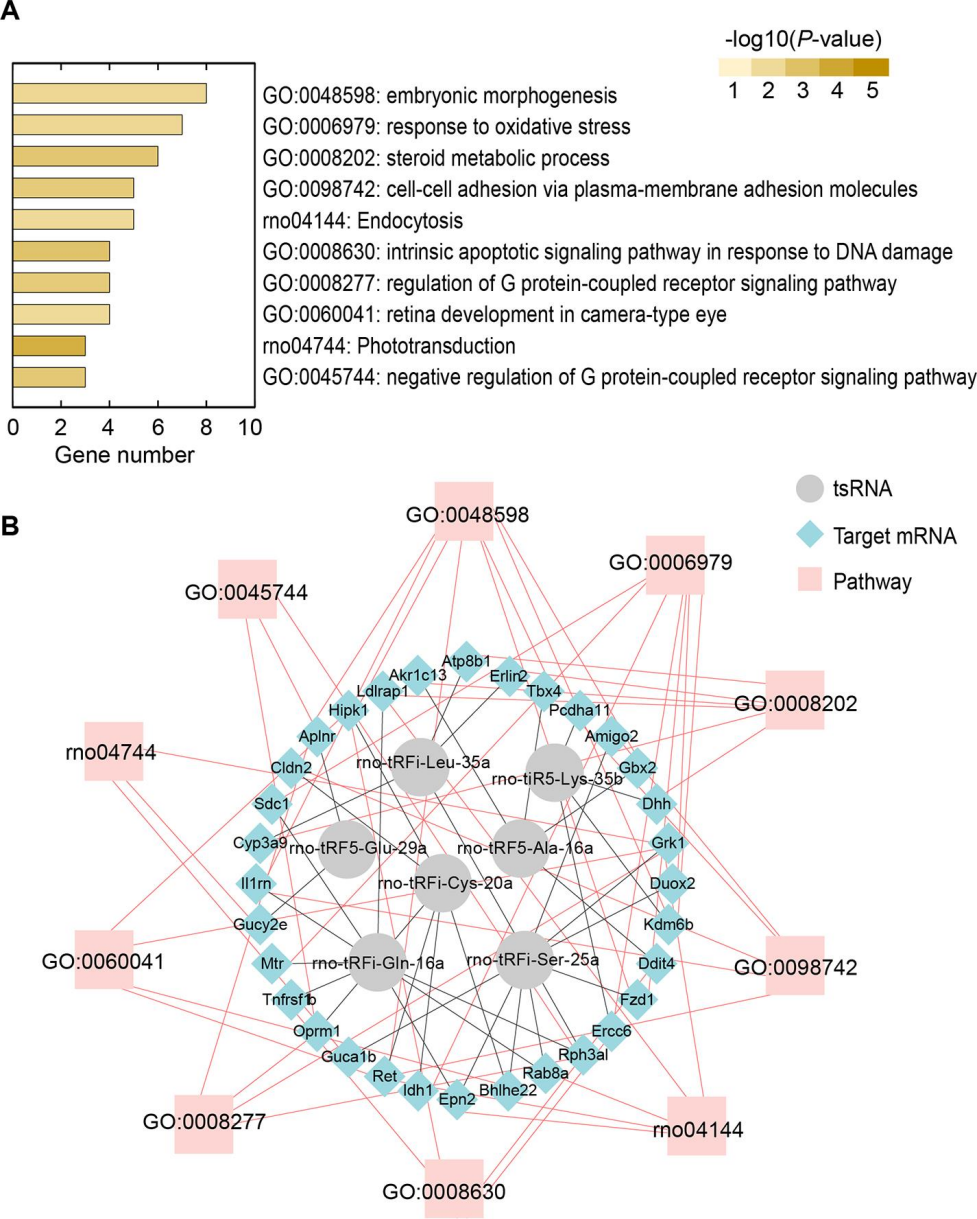
**Bioinformatics analysis revealing potential biological functions of 7 ICH-responsive tsRNAs.** (**A**) Top 10 enriched biological functions ranked by the number of enriched genes and colored by P-value are shown using the Metascape online database. (**B**) tsRNA-mRNA-pathway interaction networks. tsRNAs could target mRNA and affect their expression level in a miRNA-like manner. Then, the altered mRNAs affect the corresponding pathways to alter biological functions.

The tsRNAs could decrease target mRNA expression levels, and hence, to understand their biological functions, we conducted bioinformatics analysis to reveal the functions of the 89 significantly changed target mRNAs. In the study, we used Metascape to understand the biological functions of the 89 target mRNAs of the 7 ICH-responsive tsRNAs. Gene ontology (GO) annotations and KEGG pathway analyses were applied to show the pathophysiologic significance of these targets after ICH (Figure 6A). The major biological processes surveyed by GO annotations were embryonic morphogenesis (GO:0048598; enriched genes: Ret, Fzd1, Il1rn, Aplnr, Gbx2, Tbx4, Kdm6b, and Hipk1), response to oxidative stress (GO:0006979; enriched genes: Idh1, Sdc1, Fzd1, Duox2, Mtr, Ercc6, and Kdm6b), steroid metabolic process (GO:0008202; enriched genes: Dhh, Cyp3a9, Erlin2, Atp8b1, Akr1c13, and Ldlrap1), cell-cell adhesion by plasma-membrane adhesion molecules (GO:0098742; enriched genes: Ret, Il1rn, Amigo2, Cldn2, and Pcdha11), intrinsic apoptotic signaling pathway in response to DNA damage (GO:0008630; enriched genes: Ddit4, Tnfrsf1b, Ercc6, and Hipk1), and regulation of G protein-coupled receptor signaling pathway (GO:0008277; enriched genes: Oprm1, Grk1, Aplnr, and Rph3al). Additionally, the KEGG pathways analysis indicated that Endocytosis (rno04144; enriched genes: Ret, Epn2, Grk1, Rab8a, and Ldlrap1) was the chief signaling transduction pathways associated with the targets of the ICH-responsive tsRNAs.

**Figure 7:**
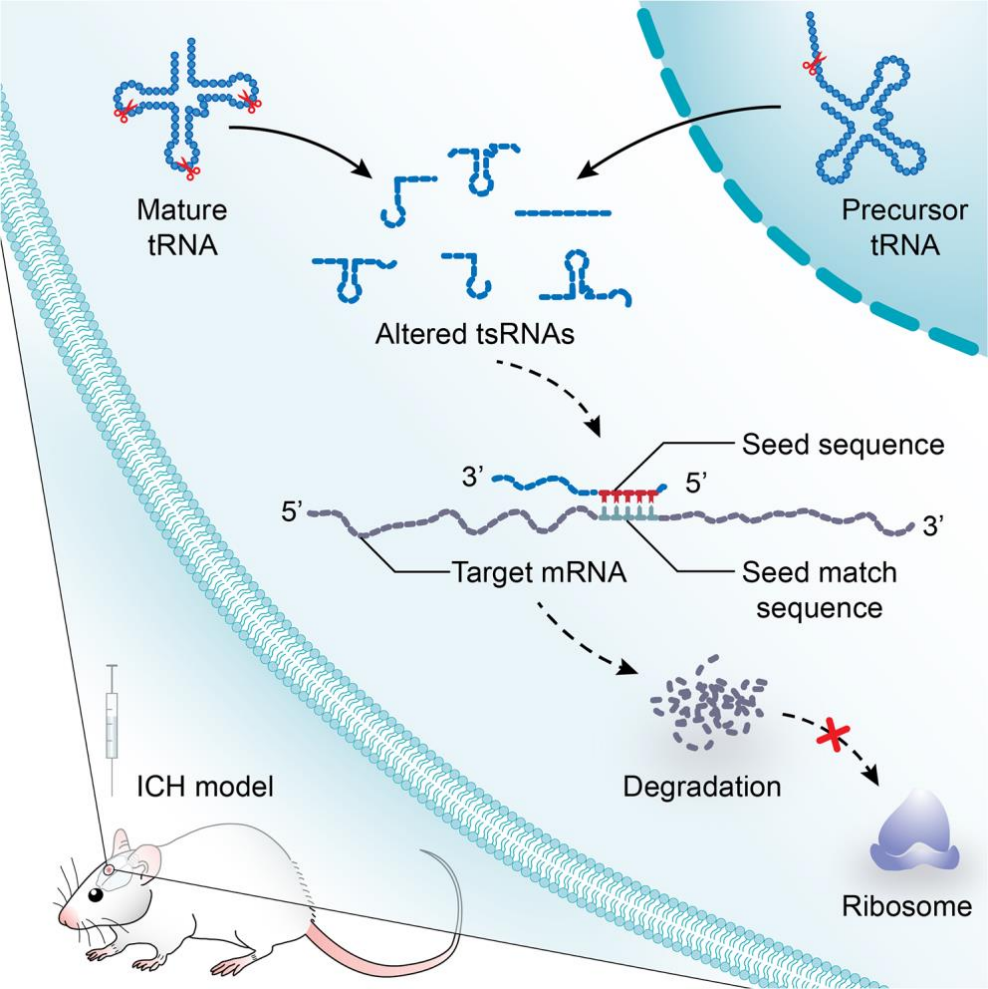
**A model illustrating the study on the potential functions of tsRNAs after ICH.** tsRNA is a novel class of short non-coding RNA cleaved from mature or precursor tRNA transcript. In a manner similar to miRNAs, tsRNAs containing some seed sequences might pair with the crosslink-centered regions of target mRNAs, leading to mRNA degradation. After ICH, the profile expression of tsRNAs is altered significantly. The subsequent effects on mRNA targets will lead to the changes of biological functions and to post-ICH pathophysiology.

Moreover, we illustrated the tsRNA-mRNA-pathway interaction networks to show the functions of the tsRNAs (Figure 6B). For instance, the 6 tsRNAs (rno-tRFi-Cys-20a targeting Ret and Hipk1, rno-tRFi-Ser-25a targeting Fzd1 and Il1rn, rno-tRFi-Gln-16a targeting Il1rn, rno-tRF5-Glu-29a targeting Aplnr, rno-tRF5-Ala-16a targeting Gbx2 and Tbx4, and rno-tiR5-Lys-35b targeting Kdm6b) regulated the 8 target mRNAs to participate in the process of embryonic morphogenesis (GO:0048598) (Figure 6B). Overall, the bioinformatics analysis indicated that the 7 ICH-responsive tsRNAs could target and modulate the 89 mRNAs, and then affect the corresponding pathways to alter their biological functions after ICH (Figure 7).

## DISCUSSION

This study systematically revealed the changes of tsRNA profiles after an experimental ICH, which may provide new insights into the pathophysiology of hemorrhagic stroke. Since functional recovery in the chronic phase is a key problem in ICH treatment [6], we chose this phase to investigate the potential regulatory functions of tsRNAs. We found that there were 7 markedly different tsRNAs (1 up-regulated and 6 down-regulated) in the rat brain after ICH compared to the sham group. Bioinformatics of tsRNA-mRNA-pathway interactions revealed the altered biological functions including response to oxidative stress, regulation of G protein-coupled receptor signaling pathway, and endocytosis. This study aimed to explore the regulating function of tsRNAs in ICH, to draw more attention from other researchers toward conducting further studies on tsRNAs.

tsRNA is an abundant class of small ncRNA whose biological roles lack sufficient understanding [16, 31]. Some previous studies have analyzed the expression profile of tRFs in rat brains of aging and cerebral ischemia, but they only concentrated on limited types of tsRNAs [20, 25]. In the present study, we systematically studied all types of tsRNAs in rat brain. The results suggest that apart from tRFs, tiRNAs are the main kind of tsRNAs in brain tissue. Although there are several common databases (tRFdb, MINTbase and TRF2Cancer) for tsRNAs including various samples from human, mouse, drosophila, etc., information on tsRNAs from rats remains absent [17, 32, 33]. Hence, the sequence results of the study may contribute to the expansion of current databases. Furthermore, as 331 tsRNAs in rat brain had been identified, we discovered that there is no unification of conventions for naming rat tsRNAs, making them unfavorable for further studies. Thus, we described a uniform naming system referring to miRNA naming conventions [27]. With this convention, a unique identifying name conveying the basics is given to each tsRNA, which benefits research in other species as well. The unification of names may be used for further studies on tsRNAs and as a reference by other researchers.

Although the biological roles of most tsRNAs remain largely elusive, several functions have been put forward. For instance, a growing body of evidence has found that several tRFs play important roles in cellular homeostasis and regulation of transposition [19, 34]. The tsRNAs are similar to miRNAs in length and structure [17, 18]. Kumar et al. found that tRF-5s and tRF-3s preferred to associate with AGO1, 3 and 4 rather than AGO2, and could interact with mRNAs in a manner similar to miRNAs [23]. The i-tRF is a new kind of tRF, and its function has not been reported clearly. However, considering the structural similarity with miRNA, i-tRFs might have similar functions as tRF-3s and tRF-5s. Additionally, the contents of tRF-5s were quite different from tRF-3s in the rat brain, suggesting functional differences between tRFs [23]. Despite differences among the types of tsRNAs, increasing numbers of studies strongly suggest that tsRNAs may function like miRNAs by interacting with complementary sequences on target mRNAs to recruit them to argonaute protein-containing complexes and to inhibit the expression or function of these targets [16, 20, 22, 23, 35–40]. Based on the miRNA-like function, several studies have predicted the targets referencing miRNA target predictors to investigate the potential biological functions of tsRNAs [16, 20], although there is no dedicated database of target prediction for tsRNAs. In Karaiskos’s study, genes with exact matches of 7mer candidate seeds to the longest annotated 3’UTR were considered potential targets. This approach is similar to that used in TargetScan [41]. Luo et al. used RNAhybrid to scan all of the transcripts for target sites with the following requirements: (i) perfect pairing between the nucleotides 2–11 of the tsRNAs and the target sites, and (ii) at most four mismatches between nucleotides 12–21 of the tsRNAs and target sites [28]. Kuscu et al. used RNA22 to search for the targets of tRF-3009a [29]. Karaiskos et al. performed seed sequence analysis by TargetScan. They generated 7-mer subsequences of tsRNAs by applying a 7-nt sliding window and shifting by one nt from the 5’ to the 3’ end, and genes with exact matches of 7-mer and 7-mer_1a candidate seeds to the 3’UTR were considered potential targets [20]. Taken together, there is no uniform algorithm approach to predict the targets of tsRNAs. The only thing the previously proposed methods have in common is that they used approaches developed for miRNA target predictors. Therefore, it is feasible in theory to predict targets of tsRNAs by different miRNA target predictors. Here, we used 3 common algorithms to predict the targets of ICH-responsive tsRNAs.

In this manner, 2510 targets were predicted simultaneously. However, there is still a need to reduce the false-positive results, considering that only about half of the predicted targets of miRNAs based on the currently employed algorithmic tools turn out to be actual targets [42]. Whether the expression levels of these potential targets were altered in ICH group compared to the sham group remained unknown. Only the differently expressed targets could be involved in the pathologic processes of ICH. Therefore, we filtered the targets using the mRNA data to find the significantly changed targets in ICH. Then, 89 significantly changed targets were chosen for subsequent analysis as we found that most of the targets (2421) were not significantly changed in ICH compared to sham. One reason is that the bioinformatic results were calculated by algorithms, which were acquired purely through bioinformatics methods. The other reason was that the targets of tsRNA could be affected by other molecules (miRNA, long non-coding RNA called lncRNA for short, etc.) as well, which might offset the regulatory functions of tsRNAs, leading to no significant changes of their targets.

To predict the functions of the tsRNA targets, we employed functional annotation tools of Metascape for GO and KEGG pathway analysis. In this study, the most enriched pathways were embryonic morphogenesis, response to oxidative stress, steroid metabolic process, cell-cell adhesion by plasma-membrane adhesion molecules, intrinsic apoptotic signaling pathway in response to DNA damage, endocytosis, and regulation of G protein-coupled receptor signaling pathway. Our previous study evaluated the functional effects of lncRNAs and mRNAs, which revealed that altered genes were enriched in the mitochondrial matrix, G-protein coupled receptor signaling pathway and olfactory transduction [30]. Interestingly, tsRNAs and lncRNAs could both regulate the G-protein coupled receptor signaling pathway, which could be attributed to the complications of the physiopathology of ICH. In most cases, the pathways regulated by tsRNAs were different from lncRNAs. This indicated that tsRNAs might play more unique roles after ICH compared to other RNAs.

Among the highlighted pathways, some pathways seemed to have little correlation with ICH, for instance, embryonic morphogenesis, cell-cell adhesion by plasma-membrane adhesion molecules, and intrinsic apoptotic signaling pathway in response to DNA damage. However, although there is almost no literature so far about the pathways involved in ICH, these pathways are closely related to the nervous system and might participate in the pathophysiological process of ICH. For instance, embryonic morphogenesis involved the development of the nervous system and might be related to the process of neuranagenesis after ICH [43]. Endocytosis is linked with microglial phagocytosis for the absorption of hematoma after ICH [44, 45]. One pathway (response to oxidative stress) was most closely related to ICH [46, 47]. Oxidative stress could cause considerable injury to the blood-brain barrier and lead to significant central nervous system pathology [48]. Moreover, oxidative stress can affect the functions of mitochondria which are mainly associated with energy metabolism disorders after ICH [49]. The pathway analysis indicated that tsRNAs might decrease target mRNA levels and then the altered mRNAs can affect the corresponding pathways to alter biological functions. We developed the tsRNA-mRNA-pathway interaction networks to visualize the functions of the tsRNAs.

To validate the tsRNA-mRNA-pathway network, we selected the most related pathway namely response to oxidative stress. Seven genes (Idh1, Sdc1, Fzd1, Duox2, Mtr, Ercc6, and Kdm6b) were enriched in that pathway. After further reviewing the functions of each gene in UniProt, we selected 5 potentially functional genes named Idh1, Sdc1, Fzd1, Duox2, and Mtr [50]. Furthermore, through the qRT-PCR results of the rat tissues and PC12 cells transfected with mimics, we focused on Fzd1. The qRT-PCR results indicated that Fzd1 was modulated by rno-tRFi-Ser-25a. Moreover, the luciferase reporter assay further revealed rno-tRFi-Ser-25a targeted Fzd1 by antisense pairing in the seed regions. Taken together, we considered that tsRNAs had potential regulatory functions in the physiopathology of ICH by regulating target mRNAs. Although this study revealed the altered expression profiles of tsRNAs in ICH rats, our study was limited to young rats only. Therefore, the functional study of tsRNAs in aged rats is the direction of our future research.

Overall, our study revealed the altered expression patterns of tsRNAs in rat brains after ICH, which might be involved in the regulation of different biological functions during the response to ICH. This information will contribute to further research on the pathogenesis and novel therapeutic targets of hemorrhagic stroke in the chronic phase. Future studies should decipher changes of expression and the function of tsRNAs in the different stages of ICH and in clinical specimens.

## MATERIALS AND METHODS

### Rat ICH models

A total of 18 adult male Sprague–Dawley rats (180-220 g) were obtained from the Laboratory Animal Centre of Central South University (CSU). All animal protocols were approved by the Committee on the Use and Care of Animals of CSU and conformed to the Guidelines for the Care and Use of Laboratory Animals. Rats were randomly assigned to the sham-operated and ICH groups [51]. In our study, we used the collagenase-induced ICH model [52]. This is a classic animal model, which is widely used in ICH study and recognized by many researchers [52, 53]. This model also mimics the hematoma expansion of continuous bleeding that occurs naturally in ICH patients [54]. ICH was induced as described in detail previously [30, 55–57]. Briefly, animals were anesthetized by intraperitoneal injection of pentobarbital (65 mg/kg). Then, the rats were fixed in a prone position on a stereotactic frame (Stoelting Co., Chicago, IL). After making a scalp incision, we drilled a small cranial burr near the right coronal suture (1.4 mm posterior and 3.2 mm lateral to bregma). Then, collagenase type VII (0.5 U dissolved in 2.5 μl saline) was slowly injected into the right globus pallidus (5.6 mm deep) at an even speed over 2 minutes. The needle was kept in place for another 5 minutes then withdrawn to prevent backflow. Finally, the burr hole was filled with bone wax, and the scalp was sutured. Sham rats underwent the same procedure but with a 2.5 μl saline infusion instead. At days 21 after the procedure, brain tissue surrounding the hemorrhagic region was harvested for study.

### Pretreatment of tsRNA*s*

Total RNAs were extracted from sham and ICH (n=3 each group) according to the manufacturer’s instruction (Qiagen, USA). The purity and concentration of total RNA samples were determined with NanoDrop ND-1000. Then, the following treatments were performed to remove some RNA modifications that might interfere with small RNA-sequencing library construction: 3’-aminoacyl (charged) deacylation to 3’-OH (hydroxyl group) for 3’ adaptor ligation, 3’-cP (2’, 3’-cyclic phosphate) removal to 3’-OH for 3’ adaptor ligation, 5’-OH phosphorylation to 5’-P for 5’-adaptor ligation, and N1-methyladenosine and N3-methylcytidine demethylation for efficient reverse transcription. All procedures were performed using the rtStar tRF&tiRNA Pretreatment Kit (Arraystar, USA) protocols.

### Library preparation and RNA sequencing (RNA-Seq)

Total RNA samples were sequentially ligated to 3’ and 5’ small RNA adapters using the NEBNext Multiplex Small RNA Library Prep Set for Illumina (New England Biolabs, USA). cDNA was then synthesized and amplified using Illumina’s proprietary reverse transcription primers and amplification primers. Subsequently, 135-170 bp PCR amplified fragments (corresponding to 15-50 nt small RNA size range) were extracted and purified from the polyacrylamide (PAGE) gel. The completed libraries were quantified by an Agilent 2100 Bioanalyzer. Libraries were mixed in equal amounts according to the quantification results and used for sequencing on the instrument. Next, the DNA fragments in the well-mixed libraries were denatured to generate single-stranded DNA molecules and loaded onto the reagent cartridge. Finally, the sequencing run was performed on Illumina NextSeq 500 system using the NextSeq 500/550 V2 kit (Illumina, USA) according to the manufacturer’s instructions. Sequencing was performed by running 50 cycles.

### Data analysis

Illumina NextSeq 500 raw sequencing read data that passed the Illumina chastity filter were used for the following analysis. Sequencing quality was examined by FastQC software. After that, the sequencing reads were 5’, 3’-adaptor trimmed, filtered for more than 15 nt by Cutadapt software. Trimmed reads were aligned to mature-tRNA and pre-tRNA sequences from GtRNAdb (http://gtrnadb.ucsc.edu/) using NovoAlign software (v2.07.11) to exactly distinguish the tsRNAs. The tsRNA expression levels were measured and normalized as tag counts per million of total aligned tRNA reads (TPM). For each tRNA sequence-based profile, the number of tRNA sequence reads could be used to estimate the expression level of each tsRNA. The statistical significance of the difference may be estimated using the Student’s *t* test. Fold changes (FC, ICH versus sham) were used for comparing two groups of profile differences. |log_2_ FC|>|log_2_ 1.5| and *P* <0.05 were considered significantly different expression levels and these ICH-responsive tsRNAs were chosen for further analysis.

### Target prediction

Increasing studies have demonstrated that tsRNAs contained some seed sequences that might match the seed regions of mRNA by antisense pairing, regulating the expression level of target mRNA in a miRNA-like manner [22–24]. The 2-7 nucleotides of the tsRNAs are called the seed sequences which could perfectly pair with nucleotides in the 3’UTR region of mRNAs. Although there is currently no database of target prediction for tsRNAs, some studies have used different algorithms to obtain possible seed sequences and targets for tsRNAs referencing to miRNA target predictors [16, 20, 28, 29]. Here, we used three common algorithms to predict tsRNA targets, namely, RNAhybrid (https://bibiserv.cebitec.uni-bielefeld.de/rnahybrid/), TargetScan (http://www.targetscan.org) and miRanda [58–60]. Through the rat database of TargetScan and miRanda, perfectly matched mRNAs were considered as the target genes. Moreover, RNAhybrid was used to access the possibility of a combination of tsRNAs and their targets (with threshold of minimum free energy <-20 kcal/mol). Finally, the targets predicted by all 3 algorithms simultaneously were chosen for further analysis. The network illustration was visualized with Cytoscape software (version 3.5.1, the Cytoscape Consortium, San Diego, CA, USA).

### Validate the predicted targets of tsRNAs with the mRNA transcriptome date

The target prediction results calculated by bioinformatics methods need to be validated [42]. Here, we used mRNA microarray data to confirm whether the expression levels of these target mRNAs were actually changed. In our previous study, we evaluated the expression profiles of the mRNAs in the same samples (ICH and sham) by microarray [30]. Consequently, the differentially expressed mRNAs (ICH versus sham, |log_2_ FC|>|log_2_ 1.5| and *P* <0.05) were used to validate the predicted targets of the tsRNAs. The mRNA targets contained in these significantly altered mRNAs were chosen as the confirmed target. Therefore, we took the intersection of the predicted targets and the altered mRNAs to obtain the validated targets of the ICH-responsive tsRNAs.

### Further validation by quantitative reverse transcription polymerase chain reaction (qRT-PCR)

The ICH-responsive tsRNAs and their mRNA targets were further detected by qRT-PCR. Reverse transcription to cDNA was achieved using rtStar™ First-Strand cDNA Synthesis Kit (3’ and 5’ adaptor; Arraystar) according to the manufacturer’s protocol. Then, qRT-PCR amplification was performed using a ViiA 7 Real-time PCR System (Applied Biosystems) and 2×PCR master mix (Arraystar). The cycling profile was as follows: incubation at 95 °C for 10 min, followed by 40 cycles of 95 °C for 10 s, 60 °C for 60 s and 95 °C for 15 s. The relative expression levels were calculated using the 2^−ΔΔCt^ method. tsRNAs were normalized by U6 and mRNAs by β-actin. The specific primers for each gene are listed in Table 1. All reactions were performed in triplicate.

**Table 1:** Primers designed for qRT-PCR validation of candidate tsRNAs and mRNAs.

**Gene name**	**Forward and reserve primer**	**Product length (bp)**
rno-tRFi-Cys-20a	F: 5’ AGTCCGACGATCAGAGCATTT 3’	49
	R: 5’ GTGTGCTCTTCCGATCTGATCT 3’	
rno-tRFi-Ser-25a	F: 5’ CCGACGATCATGGACTGCTA 3’	44
	R: 5’ CTTCCGATCTAGAGCACAATGG 3’	
rno-tRFi-Gln-16a	F: 5’ GAGTTCTACAGTCCGACGATCT 3’	47
	R: 5’ CTTCCGATCTGGATTCAGAGT 3’	
rno-tRF5-Glu-29a	F: 5’ ACAGTCCGACGATCTCCCATA 3’	57
	R: 5’ TGCTCTTCCGATCTAGGAATCC 3’	
rno-tiR5-Lys-35b	F: 5’ ATCGCCCGGCTAGCTCAGT 3’	47
	R: 5’ TTCCGATCTAGAGTCCCATGCTC 3’	
rno-tRFi-Leu-35a	F: 5’ GGATTTAGGCTCCAGTCATTTC 3’	49
	R: 5’ GTGCTCTTCCGATCTACCCA 3’	
rno-tRF5-Ala-16a	F: 5’ GTTCAGAGTTCTACAGTCCGACG 3’	49
	R: 5’ CCGATCTACTGAGCTACATCCC 3’	
U6	F: 5’ GCTTCGGCAGCACATATACTAAAAT 3’	89
	R: 5’ CGCTTCACGAATTTGCGTGTCAT 3’	
Il1rn	F: 5’ GCCTGTCTTGTGTCAAGTCTGGAG 3’	150
	R: 5’ AGGCAAGTGATTCGAAGCTGGTG 3’	
Mtr	F: 5’ CCAGCACAGAGCGTCCAAGATG 3’	161
	R: 5’ AGAGGAGCAACGAAGTCTGAGAGG 3’	
Fzd1	F: 5’ TGAAGCATGACGGCACCAAGAC 3’	110
	R: 5’ GAAGTAGCAGGCGATGACGATGG 3’	
Duox2	F: 5’ GCACAGCAGCCAGCATCTCC 3’	103
	R: 5’ GGAATGTAGCGGTTGAGGAAGGTC 3’	
β-Actin	F: 5’ ACATCCGTAAAGACCTCTATGCC 3’	223
	R: 5’ TACTCCTGCTTGCTGATCCAC 3’	

### Cell culture and transfection

PC12 cells, which are generally used as a neuronal cell line, were cultured in Dulbecco's modified Eagle's medium (DMEM; Gibco, USA) containing 5% heat-inactivated horse serum (Beyotime, China) and 10% fetal bovine serum (PAN, Germany). The cells were incubated in 5% carbon dioxide incubators at 37 °C. During the exponential phase of growth, PC12 cells were cultured in 12-well plates for transfection. The rno-tRFi-Ser-25a mimic (AUGGA-CUGCUAAUCCAUUGUGCUCU), rno-tRFi-Gln-16a mimic (TCTGGACTCTGAATCC), and the negative control (NC; UUUGUACUACACAAAAGUACUG) were obtained from RiboBio (Guangzhou, China). The transfection of mimics and NC was performed using Lipofectamine 3000 (Invitrogen, USA) at a final concentration of 200 nmol, according to the manufacturer’s instructions. All groups were performed in triplicate. After 48 hours of transfection, the transfected cells were harvested for total RNA isolation. The tsRNA-targeted genes were then measured by qPCR. The specific primers are listed in Table 1 and the protocols were as described as above.

### Luciferase reporter assay

The fragment of Fzd1 wild type (Fzd1-WT) and Fzd1 mutant type (Fzd1-mut) were cloned into the downstream site of the Renilla pmiR-RB-Report^TM^ vector (RiboBio, Guangzhou, China). With the help of Lipofectamine 3000 (Invitrogen, USA), PC12 cells were co-transfected with the above-described vectors (Fzd1-WT and Fzd1-mut) and tsRNA mimics. After 48 h post-transfection, luciferase activity was examined using the Dual Luciferase Reporter Assay System (Promega, USA) according to the manufacturer’s instructions. Renilla luciferase activity was normalized to Firefly luciferase activity for each transfected well. All groups were performed in triplicate.

### Bioinformatics analysis

Finally, Gene Ontology (GO) annotations and Kyoto Encyclopedia of Genes and Genomes (KEGG) pathway analysis were applied to assign the biological annotation of the targets, through Metascape (http://metascape.org) [61]. Enriched terms with *P*<0.01, minimum count 3, and enrichment factor >1.5 were collected and grouped into clusters based on their membership similarities. More specifically, *P-*values were calculated based on accumulative hypergeometric distribution and the enrichment factor was the ratio between the observed count and the count expected by chance.

### Statistical analysis

Results are shown as the mean ± standard error of the mean (SEM). GraphPad Prism (version 7.00; GraphPad Software, Inc., La Jolla, CA, USA) was used for statistical analysis. For normally distributed data, an unpaired Student’s *t* test was performed when the sham and ICH groups were compared. Non-parametric data were analyzed using a Mann-Whitney *U* test. *P* <0.05 was regarded as statistically significant.

## Supplementary Material

Supplementary Figures

Supplementary Table 1

Supplementary Table 2

Supplementary Table 3
