# Supplementary material for: Integrative analysis of transcriptomes highlights potential functions of transfer-RNA-derived small RNAs in experimental intracerebral hemorrhage
Source: Aging (Albany NY). 2020 Nov 16;12(22):22794–813. doi: 10.18632/aging.103938 (PMC7746353; doi:10.18632/aging.103938)
Supplement: Supplementary Figures [file aging-12-103938-s001..pdf]

SUPPLEMENTARY FIGURES

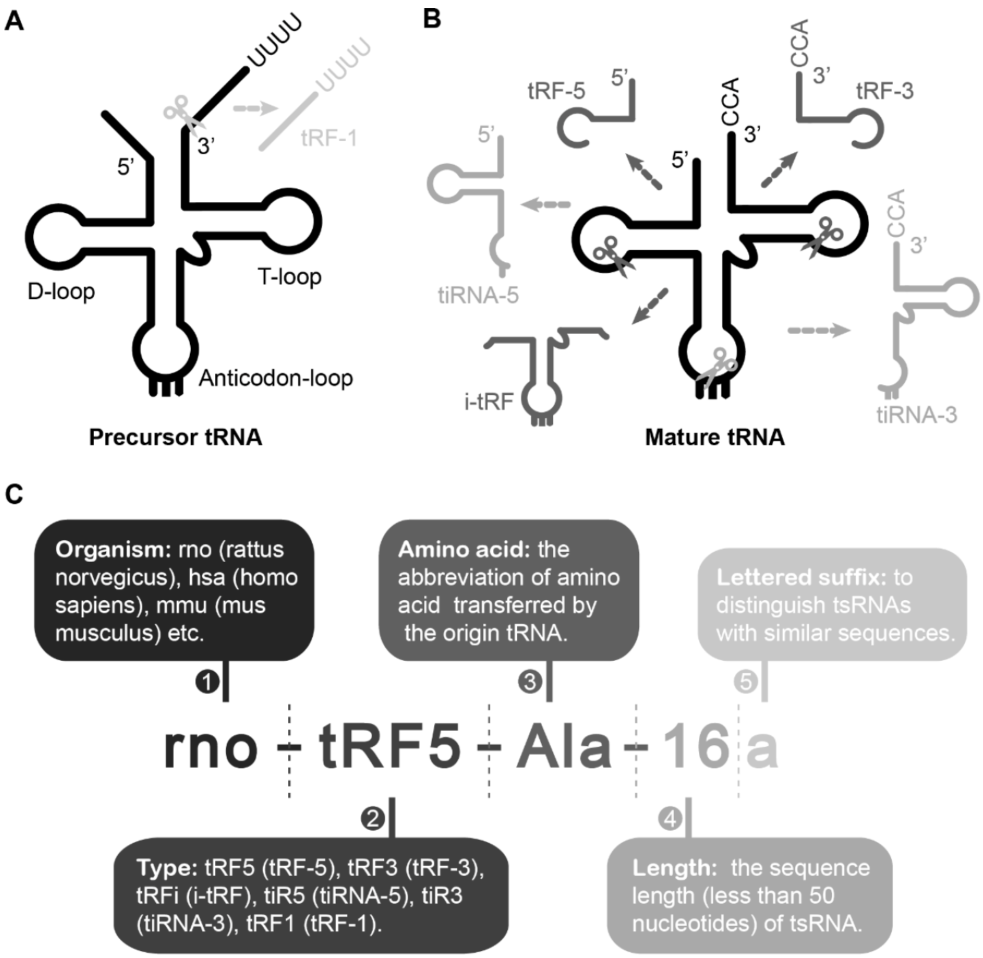

Supplementary Figure 1. Classification and a uniform naming system of tsRNAs. The 6 types of transfer-RNA-derived small RNA (tsRNA) that differed in the cleavage position, one from the precursor tRNA (**A**) and five from the mature tRNA (**B**). A uniform system for tsRNA annotation in 5 steps: organism, type, amino acid, length and lettered suffix (**C**). The first three letters signified the organism. The second part represented the type (tRF5 indicates tRF-5; tRF3, tRF-3; tRFi, i-tRF; tiR5, tiRNA-5; tiR3, tiRNA-3; and tRF1, tRF-1). The third was the abbreviation of amino acid transferred by the origin tRNA. Although one tsRNA might be derived from several tRNAs, these tRNAs usually transferred the same amino acid. Consequently, it was a one-to-one match between the tsRNA and amino acid. Rarely, tRNAs (chr17.trna3927-ValCAC, chr17.trna3928-ValCAC, and chr10.trna7847-AlaAGC) from which rno-tRF5-Val-23b arose, transferred Valine (Val) and Alanine (Ala). In this case, we used the first tRNA ranked by the identifying number to represent the origin tRNA. Fourthly, the number denoted the sequence length. Finally, lettered suffixes were used to distinguish highly similar sequences. For instance, rno-tiR5-Gly-31a (GCATTGGTGGTTCAGTGGTAGAATTCTCGCC) and rno-tiR5-Gly-31b (GCATGGGTGGTTCAGTGGTAGAATTCTCGCC) were nearly identical while slightly different at the fifth nucleotide in the sequence. With the uniform system, a unique identifying name was given and could convey the basic information of tsRNAs.

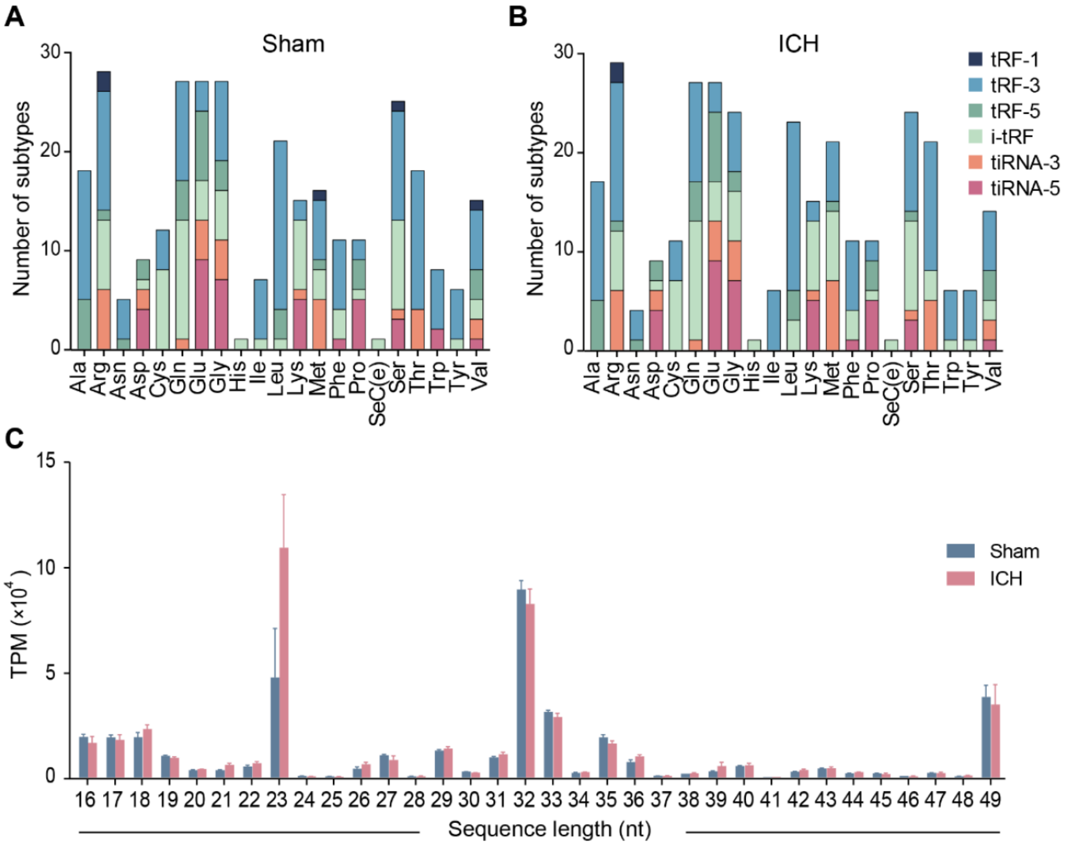

Supplementary Figure 2. The information of tsRNAs in each group. Stacked plot for all subtype of tsRNAs of the sham (**A**) and ICH (**B**) group clustering by the anticodon of the tRNAs. The X axis represents the tRNAs with the same anticodon, and the Y axis show the number of all subtype tsRNAs derived from the same anticodon tRNA. The bar with color represents the number of each subtype tsRNA. (**C**) The lengths of tsRNAs were concentrated in the range of 16-23, 29-36, and 49 nt. TPM indicates tag counts per million of total aligned tRNA reads, representing tsRNA expression levels.
